# Exploring the perspectives of clinical professionals and support staff on implementing supported self-management for asthma in UK general practice: an IMP^2^ART qualitative study

**DOI:** 10.1038/s41533-017-0041-y

**Published:** 2017-07-18

**Authors:** Susan Morrow, Luke Daines, Sharon Wiener-Ogilvie, Liz Steed, Lorna McKee, Ann-Louise Caress, Stephanie J. C. Taylor, Hilary Pinnock

**Affiliations:** 1 Asthma UK Centre for Applied Research, Usher Institute for Population Health Sciences and Informatics, University of Edinburgh, Edinburgh, UK; 2 NHS Education for Scotland, Edinburgh, UK; 3 Centre for Primary Care and Public Health, Barts and The London School of Medicine and Dentistry, Queen Mary University of London, London, UK; 4 Health Services Research Unit, University of Aberdeen, Aberdeen, UK; 5 School of Nursing, Midwifery & Social Work, University of Manchester, Manchester, UK

## Abstract

Despite an overwhelming evidence base, supported self-management of asthma is poorly implemented into routine practice. Strategies for implementation must address organisational routines, as well as provide resources for patients and training to improve professionals’ skills. We aimed to explore the priority that primary care practices attach to asthma self-management, to describe their existing asthma management routines, and to generate innovative implementation strategies. We recruited 33 participants (23 general practitioners; seven nurses; three administrative staff) from 14 general practices. The 12 interviews and three focus groups were transcribed, coded and analysed thematically. Supported self-management was largely a nurse-led task within clinic-based annual reviews. Barriers included poor attendance at asthma clinics, lack of time, demarcation of roles, limited access to a range of tailored resources, and competing agendas in consultation, often due to multimorbidity. Suggestions for initiatives to improve the provision of supported self-management included emphasising the evidence for benefit (to influence prioritisation), improving teamwork (including team-based education), organisational strategies (including remote consulting) which need to fit within existing practice routines. Technology offers some potential solutions (e.g., improved templates, ‘app’-based plans), but must be integrated with the practice information technology systems. Building on these insights, we will now develop a theoretically-based implementation strategy that will address patient, professional, and organisational buy-in, provide team-based education and offer a range of practical options and tools, which can be adapted and integrated within existing routines of individual practices.

## Overcoming the organisational barriers to implementing asthma self-management

Understanding the routines of primary care practices can suggest strategies to implement supported self-management in general practice. Supported self-management of asthma including provision of individual action plans improves patient health and reduces the burden on healthcare services, but is not well implemented in routine practice. As part of a large-scale programme to implement self-management into UK general practice, Hilary Pinnock at the University of Edinburgh and co-workers conducted interviews and focus groups with 33 participants from 14 general practices to explore the organisational routines that hinder or enable professionals to provide support asthma self-management. Poor attendance at asthma clinics, demarcation of roles, lack of time and limited access to tailored resources were identified as specific barriers. Improvements suggested included improved teamwork between doctors and other medical healthcare professionals, comprehensive training, and improvements to IT systems.

## Introduction

Asthma is common (334 million people worldwide), responsible for substantial morbidity and an increasing burden on healthcare services globally.^1^ With good management most people with asthma should be symptom-free most of the time.^2, 3^ Despite this, in the UK, there are over six million primary care consultations, and 100,000 hospital admissions each year, at an estimated cost of £1billion a year.^4^ Each year, 1200 people die from asthma—the majority of these deaths are preventable with timely (self) management.^5^

There is a substantial body of evidence that supported self-management, including a personalised asthma action plan (PAAP) and supported by regular professional review, enables people to adjust their treatment in response to worsening symptoms, with over-whelming evidence that this improves day-to-day asthma control, reduces the risk of asthma attacks and use of healthcare resources.^6, 7^ Despite provision of supported self-management for all people with asthma being a guideline recommendation for a quarter of a century,^2, 3, 8^ surveys internationally suggest that fewer than a third of people own an action plan.^9–12^ Effective implementation of supported self-management^13, 14^ requires a whole systems approach in which the role of the organisation in facilitating professionals’ engagement and ensuring support for patients is fundamental.^15^ Frameworks (e.g., considering time, resources and people in the context of routinisation theory^16, 17^) for describing and changing organisational behaviour emphasise the need to understand, and integrate change within existing practice routines. Flexible implementation strategies adapted to individual practice routines and context,^18, 19^ may thus ‘nudge’ practices towards potentially sustainable change.^20^

As part of the IMP^2^ART (IMPlementing IMProved Asthma self-management as Routine Treatment) programme of work we are developing a whole systems implementation strategy for embedding optimal supported asthma self-management within routine care. We here report a qualitative study which aimed to explore how primary care practices prioritise asthma self-management, to describe their existing asthma management routines and the barriers and facilitators, and to generate innovative practical strategies to implement supported self-management for children over 5 years and adults, and how they may be optimally introduced.

## Results

### Participants

Overall 33 professionals **(**23 general practitioners (GPs), seven asthma nurses, and three administrative staff) provided 12 interviews and three focus groups, representing perspectives from people working in 14 general practice healthcare settings. Table 1 provides details of their characteristics.

All focus groups took place face to face and lasted between 25 and 60 min The interviews were completed in a mean of 30 min (shortest 15 min). Despite the busy primary care context, field notes confirm good rapport and engagement with the interview topic in the five telephone and seven face to face interviews.

**Table 1:** Sources of data

Group	Regions
Practice Nurse (N1-N5)	Forth Valley, Central Scotland, Lothian and Kent
General Practitioner (GP1-4)	Leeds, Yorkshire, Kent and Lothian
Focus Group 1 (FG1) (7 GPs and 2 practice nurses)	Primary Care Medical Practice, Lothian
Focus Group 2 (FG2) (7 sessional GPs)	Sessional GPs, London
Focus Group 3 (FG3) (5 GPs)	Primary Care Medical Practice, Kent

### Summary of the main themes

**Fig. 1:**
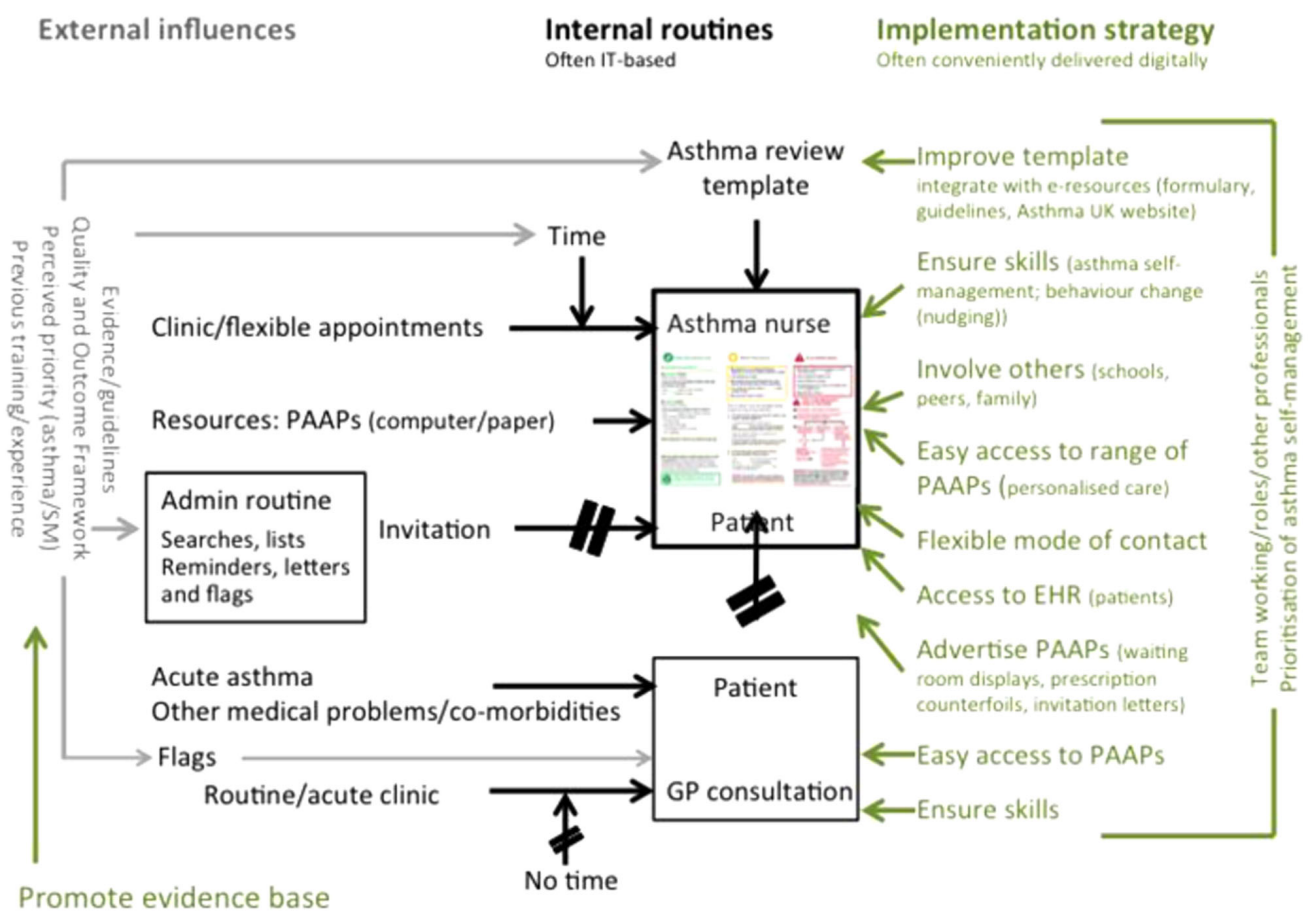
Schema for a ‘typical’ routine for provision of self-management and personalised asthma action plans in general practices. The *black boxes and lines* in the schema illustrate a typical routine, described by doctors, nurses and practice staff, for providing asthma reviews and supported self-management. The *grey text and lines* illustrates the external influences that influence the priority attached to asthma care and supported self-management or which determine organisational arrangements. The *green text and lines* are suggestions made by the participants that they perceive might help overcome the challenges. *PAAP* Personalised Asthma Action Plan

Three main themes are described, related to our objectives; (i) the priority attached to asthma self-management and the external influences that determined priority and organisation, (ii) internal routines and the barriers and facilitators to providing supported self-management, and (iii) suggestions for an implementation strategy. The findings are presented under these headings, and a schema of the typical practice routine for supporting self-management is provided in Fig. 1.

#### Priority and the external influences

Supporting asthma self-management was typically described as in the top 5–10% of priorities (but usually behind diabetes, heart failure and dementia); nurses tended to prioritise it higher than GPs. Discussion of the evidence led to suggestions that ‘*maybe it should be higher*’?

*“Personally asthma is very high up my list”* (Nurse3)

*“I think we’ve got probably bigger priorities in terms of diabetes, dementia, general care of polypharmacy in the elderly”* (GP2)

The Quality and Outcome Framework (QOF) (A pay-for-performance scheme which incentivises annual asthma reviews with guidance that this should include provision of an action plan),^21^ national guidelines,^2^ and the competing demands of other clinical conditions influenced the priority given to asthma and self-management.

*“We’re still using QOF… so I guess that directs what the boxes are because you’re ticking the QOF ones”* (Nurse3)

*“I think* [the priority] *for us locally* [is], *our diabetes management, although it was good in the practice it wasn’t good in other areas, so because it was a weakness you’ve got to focus on it… … I think our asthmatics are better managed than our COPD patients*” (GP1)

#### Internal practice routines and barriers to provision of supported self-management

##### Professional roles and delegation

Professionals identified the provision of asthma self-management as being predominantly a nurse role undertaken within a face-to-face clinic consultation, with GPs typically feeling relatively unfamiliar with the practicalities of providing asthma action plans.

*“We have a very skilled nurse team who do the routine preventative stuff”* (GP3)

GPs tended to have a minimal role in supporting self-management, instead seeing patients during asthma exacerbations or if patients attended for other reasons. Time precluded providing self-management during such consultations, so GPs flagged up patients who had not had an annual review and/or required self-management support and referred to the nurse.

“*So I suppose generally what happens is for adults and children they will come and see us when it’s bad and then we will often initiate new treatment or change their current inhalers or something to deal with that particular exacerbation. And then we’ll say to them please come back and see the nurse*” (GP4)

Nurses in particular recognised, and voiced frustration, at the issues associated with demarcation of roles within the practice.

[Interviewer: Are there other members of staff that are involved in arranging these reviews and seeing patients?] *“And that’s a no and that’s where it gets a bit frustrating for me…. don’t feel I have proper support from one of my GPs and they don’t seem to have an interest which is a bit frustrating.”* (Nurse1)

GPs recognised the problems associated with delegation but felt unable to address this, at least in part because there were so many other priorities to think about.

*“It’s OK talking in terms of delegating to the asthma nurse but the patient doesn’t want to come back or they promise to come back but they don’t”* (GP2)

“*When you’re in a sort of busy walk-in surgery you deal with the acute situations, but you don’t have that time that you can say ‘While we’re here, let’s go back and talk about how do you use your spacer?’ and ‘What would you do if you got a cold?’ and ‘You know what do you do during the winter and in the summer?’“* (GP1)

Notwithstanding the practical difficulties, the GPs in FG2 were very supportive of the concepts of tailored, supportive self-management:

*“I think the important thing is not to be paternalistic and didactic and educating but to find out what they* [the patients] *understand about their condition, what they don’t know from that and then to support them in that way”* (FG2 GP6)

*“And ultimately from first principles, it should be education from the time you make the diagnosis and meet the patient.”* (FG2 GP2)

*“I think it’s about health beliefs that they have … So like what you were saying about what their agenda and goals are”* (FG2 GP3).

##### Difficulty engaging patients

There was general recognition that patients wanted information about managing their asthma: *“a lot of people will say…‘if only someone had explained that to them’”* (N5 interview). Despite this many professionals felt that the patients themselves were the barrier, expressing frustration that engaging with, and encouraging them to attend for routine asthma reviews was a challenge.

*“Trying to get them in for their annual review is fun. A lot of them don’t want to come in.”* (Admin1)

*“The understanding of the patient is the barrier, I mean, to get through to them.”* (FG2 GP3)

*“… well actually these people have made the choice, we’ve done as much as we can, we’ve sent them information, we’ve tried to phone them, we’ve tried to engage with them, we’ve tried to opportunistically engage with them…. but if they choose to disengage, it’s their choice. We’re not a nanny state.’* (Nurse2)

Reasons for non-engagement were identified as patients being in denial, or not viewing their asthma as serious despite *“living in a state of not being properly controlled”* (GP4). Another common theme was the limited range of resources making it difficult to find action plans and information tailored to individual patients.

*“You know I think it’s quite common for asthma patients to kind of bury their head in the sand and avoid coming for asthma review.”* (Nurse4)

*“The problem is …. if they aren’t going to the hospital frequently or are on high dose steroids, they see their asthma as being very mild and therefore it isn’t something that should attract their attention.”* (Nurse5)

“*A lot of the wording on the management plan is either not really relevant to some patients, or it’s quite wordy and I often find the patients don’t really engage with what is actually written on it*” (Nurse3)

##### Organisational barriers of time, resources and multimorbidity

Patients were invited for review annually, with some practices setting times for asthma review clinics whereas others offered more flexibility. Most practices allocated 20 min for a review, with some nurses expressing a wish for longer appointment times.

*“Asthma register…recall them on a regular basis so everybody is recalled yearly” (*Nurse2)

“*Once a week I have an asthma clinic which is actually Wednesday afternoon where I just solely see asthma patients*” (Nurse4)

*“… the idea is that they can then make an appointment for a time that suits them…. we’d rather get them in than not at all.”* (Nurse1)

The annual review was largely structured around computer templates which had the potential to promote and facilitate provision of an action plan. In contrast poor integration with information technology (IT)-systems, alert fatigue, duplication of effort and too many tick boxes were also identified as barriers:

“W*e have a template that we would go through, symptoms, treatment, their general asthma control … as well as checking that they have a written asthma plan*” (FG1 Nurse1)

*“It’s duplication…you’re basically having to fill in two templates and free text“* (Nurse5)

*“… it’s* [flags on the computer screen] *so frequent that you just ignore it… … Every patient virtually has got some prompt”* (GP3)

*“Far too busy, too busy… tick, tick, tick*—*and then let’s get on with seeing the patients.”* (FG2 GP5)

##### Practical challenges

Practical challenges included budget limitations, limited time for dealing with complex problems, lack of resources, language and cultural barriers, and action plans that were not sufficiently adaptable or did not print well in black and white.

“*You could have an asthmatic who smokes, who is overweight, have BMI of over 30.. how do you fit that into an asthma review?”* (GP4)

“*We don’t actually have asthma action plans to my knowledge in other languages so that would be a challenge*” (Nurse2)

“*They send us something which is really useful and you think ‘yeah, I’ll print that’ and then you think ‘I can’t because I haven’t got a colour printer’*” (GP4)

*“While you’re here, I notice that we haven’t reviewed your asthma. I’m already running 40 min late”* (FG3 GP1)

#### Implementation strategy and potential solutions

Professionals suggested a number of practical strategies (including education and team-based training, personalisation of resources and mode of consultation, digital solutions) for implementing supported self-management and how they may be optimally introduced.

##### Education and training

Providing practitioners with skills and confidence was a priority, but needed to include GPs, nurses, administrative staff, and consider the possibility that practitioners outside the practice (local pharmacists, health educators, school nurses, community staff) might contribute to supporting self-management. The importance of involving the team was emphasised. Practical barriers were discussed (such as ensuring ‘back-fill’ to enable practitioners to attend training).

*“I think what would be really good would be to have more education for the clinicians who are actually managing the clinics because if they have more expertise and more understanding then so will the patients” (Nurse 2)*

*“Yeah I think your plan to do a medical update is great so we’re all singing from the same hymn sheet, we’re not all doing different things at different places*” (FG1 GP2)

*“It needs to be the receptionist getting the repeat prescriptions in as well as the pharmacist and everybody else really, doesn’t it?”* (FG2 GP6)

*“It is really difficult to release nurses from practice. It’s difficult to find backfill.”* (GP4)

##### Personalisation

Customising the approach to the individual patient and their clinical situation was suggested as a means of engaging patients and was important to many interviewees (e.g., access to a range of PAAPs, alternative modes of consultation);

*“I don’t think it would be right to have just one way of delivering a management plan I think you can have options at your disposal*” (Nurse5)

*“… an asthma plan with graphics …. You could give it to anybody that doesn’t speak English, it’s all pictures”* (FG2 GP5)

*“I do think an app is good and probably more of the population would use that. The older patients … they often prefer the paperwork to anything virtual”* (Nurse4)

*“Skype or anything like that would be ideal and young people might respond better to that”* (Nurse 3)

##### Digital solutions

Digital solutions were a very common theme, with suggestions about improving asthma review templates, facilitating access to a range of action plans, ‘apps’ for monitoring asthma, and innovative ways of using patient accessible parts of the Electronic Health Record as a repository for completed action plans. The main caveats were that digital options were not suitable for everyone, had to be intuitive for the user, and integration with practice IT systems was crucial to avoid duplication of effort.

*“I think most practice nurses use the templates and they’re very, very helpful as an aide memoire”* (Nurse2)

*“I think it’s* [computer resources] *got to be really, really user friendly and quite intuitive for people to do it. You just don’t have the time or you haven’t got that brain space to input anything that’s tricky”* (GP1)

*“I think it’s fine to have an app but it has to be one that goes with the clinical system, otherwise we’re duplicating”* (FG2 GP5)

## Discussion

This study aimed to explore the priority that practices attach to asthma self-management, to describe their existing asthma management routines, and to generate innovative implementation strategies.

### Main findings

In UK general practice, supported asthma self-management is largely a nurse-led task within clinic-based annual reviews. Barriers to provision of self-management include poor attendance at asthma clinics, lack of time, demarcation of roles, limited access to a range of resources, and competing agendas in consultations, often due to multimorbidity. Suggestions for initiatives to improve the provision of supported self-management include emphasising the evidence for benefit (to influence prioritisation), improving teamwork (including team-based education), engaging patients and adapting organisational strategies (including remote consulting) all of which need to fit within existing practice routines. Technology offers some potential solutions (e.g., improved templates, app-based plans), but with the caveat that it must be integrated with the electronic health record and existing IT systems.

### Strengths and limitations of this study

The professionals we interviewed for this study represented the views of a range of different general practice healthcare settings across the UK. We strove to obtain a wide range of views and achieved data saturation, however we acknowledge that a different sample may have presented different views and that the results from this research may not be applicable to healthcare settings outside UK.

Researchers’ attitudes influence design, data collection and analysis of qualitative studies;^22^ however, we worked with our multidisciplinary professional team and lay advisors to develop the topic guide and to ensure a balanced interpretation of the data

### Interpretation of findings in relation to previously published work

Our previous systematic meta-review of supported self-management concluded that effective implementation strategies were multifaceted and multidisciplinary; engaging patients, training and motivating professionals within the context of an organisation that actively enabled self-management.^15^ Our findings support the need to address each of these elements and add to previous research by offering practical insights into how best to implement strategies that will improve the provision of supported self-management while fitting in with existing routines and organisational constraints.

Engaging patients in attending (face-to-face) reviews was perceived as challenging; and the clinicians were balancing multiple clinical and organisational priorities. The organisational routines described to us identified practical barrier of lack of time and resources, and the central importance of the electronic health record (e.g., asthma review templates). An implementation strategy will need to address all these issues, within the context of the existing routines.

Routinisation theory holds that healthcare organisational life is highly structured around devices such as time, resources and people.^17^ We have identified all of these key devices as involved in provision of supported asthma self-management in general practice. Limited time in complex consultations, was a problem for all professionals but particularly for GPs whose acute consultations were already fully occupied with the presenting problem(s). Time may be allocated to supporting self-management in nurse-led routine reviews but, despite reminders, many patients did not attend a clinic-based consultation. Alternative modes of contact, such as telephone consultations,^23, 24^ Skype, or e-mail may help to address non-attendance. Although some of the templates that nurses used in reviews enable an action plan to be downloaded, there was concern that a wider range of options was needed to enable personalisation.

The delegation of asthma care to nurses was described nearly a decade ago,^25^ and the importance of communication highlighted as ensuring that team members were aware of (and could therefore support) each others’ roles. We found continuing demarcation of roles—self-management support was typically devolved to the practice nurse but not always with the necessary mutual understanding of the role that could have supported teamwork. This resulted in deskilling of GPs and marginalisation of asthma nurses from broader practice decision-making, prioritisation and management of asthma. GPs recognised the demarcation but, despite being aware of the importance of patient-centred care, hadn’t addressed the problem at least in part because there were so many other priorities to think about.

### Implications for future research, policy and practice

We did not detect previously reported scepticism about the effectiveness of asthma self-management in primary care,^26^ but competing demands were challenging, both in the context of managing complex consultations for individuals with multimorbidity, and at the practice level when prioritising services for different equally important conditions.

We have identified specific initiatives that could improve the routines around provision of supported asthma self-management. Greenhalgh et al. stated that in order to develop and refine organisational routines we need to pay attention to three domains; structuring devices, people and organisational learning.^17^ We have identified the key elements for each of these domains; structuring devices, such as provision of a range of action plans (including digital options^27^) and improved clinical templates, the key people involved (nurses, GPs and administrative staff) and the need for additional training, and the importance of engaging the whole team in developing and adapting the strategy for implementing self-management.

## Conclusions

Provision of supported asthma self-management in UK primary care is typically nurse-led within clinic-based annual reviews. Barriers included time, poor attendance at clinics, limited options to enable personalisation, multimorbidity and competing agendas in consultations. Technology offers some potential solutions, but needs to be integrated into the practice systems. Team-based training to enable a consistent approach between professionals, improve skills and understanding of delegated roles, and addressing barriers that prevent prioritisation of asthma were important.

Building on these insights, we can now develop a theoretically-based implementation strategy that will address patient, professional, organisational buy-in, provide team-based education and offer a range of practical options/tools, which can be adapted and integrated within existing routines of individual practices.

## Methods

We undertook semi-structured interviews and focus groups with professionals and administrative staff from general practices between March 2016 and August 2016. The study had ethical approval from South Central—Berkshire Research Ethics Committee (ref. 16/SC/0024) and was performed in accordance with NHS research management approval from NHS Lothian (ref. 2016/0031).

### Sampling and recruitment

E-mail invitations to participate in a single focus groups or interviews were distributed to GPs, practice nurses and administrative staff from ten general practices approached because of their diverse practice demography (urban and rural) and organisation (specifically including different computer systems: EMIS, Vision and SystmOne) from around the UK (Grampian, Lothian, Yorkshire, Leeds and Kent), and, to broaden perspectives, to members of an NHS Education Scotland asthma nurse group and a South London sessional GP group. The practices had previously provided routine data for service improvement purposes, but were not known to the study team. All participants provided written informed consent.

### Data collection

We offered participants the choice of interviews (either by telephone or in person at their work place) or focus group (within the practice or at an external venue). At the invitation of one practice, the researcher attended the practice for a whole day, recruiting and interviewing members of staff during breaks. Interviews (up to 50 min) and focus group (up to 1 h) were conducted by S.M., a trained researcher with a background in midwifery, with H.P. to support the focus group. The focus group with sessional GPs was conducted by H.P. and S.T., both academic GPs with an interest in provision of asthma care and self-management. Recruitment continued until data saturation from our sample of practices in respect of our key objectives had been reached. Field notes were made, and interviews and focus group were audio recorded and transcribed verbatim. The transcripts were not returned to the participants for comment.

### Topic guides

Topic guides were used to frame discussions. These guides were informed by theories of routinisation,^17^ piloted and developed in discussion with the multidisciplinary research team which included a lay member with asthma, GPs, qualitative researchers, health economist, and health psychologist. They were refined iteratively during the process of data collection. Interviews and focus group explored existing routines for supporting self-management, barriers to the provision of supportive self-management and practical strategies that could overcome barriers and facilitate change to improve implementation. The potential ‘buy-in’ for implementing self-management from the perspective of stakeholders was also explored. The over-arching focus was on identifying potential solutions to challenges; exploring barriers was used as a means of identifying ways to overcome the problems. The original detailed topic guide and a later, iteratively developed version are presented in Supplementary Appendix 1.

### Data analysis

Analysis was iterative, guided by the research questions, and key theoretical concepts related to implementing complex interventions.^17^ The researcher who had undertaken data collection immersed herself in the data, reading and re-reading transcripts in order to identify emergent categories and themes. A coding framework was drawn up in discussion with the multidisciplinary research team. The data were then coded using NVivo 8 software and the emergent coding framework was applied systematically to the entire content of the focus group and interviews. Changes and additions to coding were made to the coding framework as required, and in consultation with the multidisciplinary research team. A sample of interviews and focus group was coded independently by second coders (L.S., S.W.O. and S.T.) for comparison. The primary and secondary codes derived from the data are presented in Supplementary Appendix 2.

### Interpretation

Interpretation was informed by key implemention theories (e.g., practice theory,^16^ routinisation theory^17^) which focused attention on structuring devices, people and organisational learning. Emerging findings were presented to the multidisciplinary steering group including all the grantholders and a member of the patient and public involvement group in order to aid interpretation and obtain a wider perspective on the implications for development of an implementation strategy.

### Data availability

We do not have consent to share interview or focus group data. Further information may be available from the corresponding author.

## IMP^2^ART team

The members of the IMP^2^ART team are Audrey Buelo, Luke Daines, Nicola McCleary, Brian McKinstry, Susan Morrow, Hilary Pinnock, Aziz Sheikh, Sharon Weiner-Ogilvie (University of Edinburgh); Sandra Eldridge, Chris Griffiths, Chris Newby, Liz Steed, and Stephanie Taylor; (Queen Mary University of London); Neil Wright (University of Oxford; Anne-Louise Caress (University of Manchester); Elisabeth Ehrlich (Asthma UK Centre for Applied Research); Bethan Haskins (Canterbury and Coastal Clinical Commissioning Group); Rob Horne (University College London); Steven Julious (University of Sheffield); Lorna McKee (University of Aberdeen); Amanda Andrews, Monica Fletcher (Education for Health); Ceri Phillips, Deborah Fitzsimmons (University of Swansea); Francis Appiagyei, Tanith Hjelmbjerg, David Price, Dermot Ryan, Derek Skinner, Erica Vince-Lawer (Optimum Patient Care).

## Electronic supplementary material

Appendix 1. Detailed topic guide

Appendix 2. Coding framework
