# Supplementary material for: Exploring the perspectives of clinical professionals and support staff on implementing supported self-management for asthma in UK general practice: an IMP2ART qualitative study
Source: NPJ Prim Care Respir Med. 2017 Jul 18;27:45. doi: 10.1038/s41533-017-0041-y (PMC5515882; doi:10.1038/s41533-017-0041-y)
Supplement: Supplementary file 1 — Appendix 1. Detailed topic guide [file 41533_2017_41_MOESM1_ESM.docx]

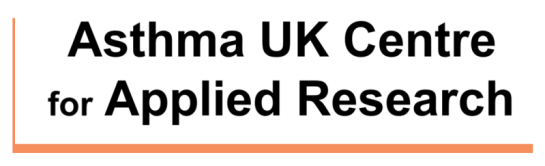

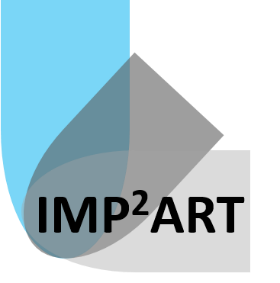


IMP^2^ART

IMPlementing IMProved Asthma self-management as Routine Treatment

**Detailed topic guide**

Version 1: 8^th^ December 2015

| **Clinicians and practice staff**  Note: the topic guide will evolve iteratively as themes emerge, with later focus groups and interviews specifically exploring emerging themes and ideas | |
| --- | --- |
| **Opening question** | **Key prompts and topics for further exploration** |
| What does the concept of supported ‘self-management’ mean to you? | Terminology?  Education/information?  Enabling patients to be responsible for adjusting treatment?  Advice on recognising, managing acute attacks? Action plans?  Advice on living with asthma? |
| Establish what supported self management means to group/interviewee but if they have a different understanding, need to establish a common understanding before moving to the next question. | |
| How does self-management fit into the routines of your practice?  How viable is supported self-management? | What is the current policy in your practice? Routines?  Whose responsibility? Do they necessary skills/training?  Role of others in team? (Are others/should others be involved?)  What resources do they have/use? (time/available PAAPs)  What would make it more viable/less viable? |
| On a practical level what are the barriers to self-management?  How could these be overcome? | Perceptual? Waste of time? Only suitable for some people?  No action plan (or can’t find them)? Worried about patients having emergency supplies of drugs?  No time/resources to provide the (accessible/flexible) support? |
| How is self-management supported? | Professional contacts? Routine reviews? Acute consultations? Hospital discharge?  Mode of access (face-to-face, telephone, text, on-line)  Repeat prescriptions? |
| What practical strategies might help implement self-management?  How would these fit in with the routines of the practice? | Computer reminders  Easier access to action plans (Format? Digital?)  ‘Living with asthma’ information (or referral mechanism?)  ‘Advertising’ action plans (e.g. on repeat prescriptions, review invitation letters, practice leaflets, posters/electronic display boards)  Access to (better) information? Trigger data eg pollen count, viral load  Regular monitoring? Cloud-based records? Patient access to EHR?  Flexible access to professional advice? Telephone? E-mail? Helpline? |
| What do you think of these ideas? | We will introduce suggestions from literature, and as the data collection and analysis proceeds, from previous focus groups and interviews, professional/manager focus groups and interviews. |
| How keen would you be to see supported self-management implemented as a normal way of working? | Any more ideas about how this could be achieved within the routines of primary care? |


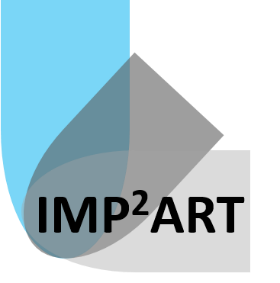

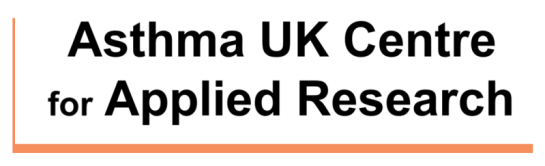


IMP^2^ART

IMPlementing IMProved Asthma self-management as Routine Treatment

IMP^2^ARTIMPlementing IMProved Asthma self-management as Routine Treatment

**Detailed topic guide: Working Copy**

| **Time** | **Opening question** | **Key prompts and topics for further exploration** |
| --- | --- | --- |
| <5 mins | What does the concept of supported ‘self-management’ mean to you? | Terminology?  Education/information?  Enabling patients to be responsible for adjusting treatment?  Advice on recognising, managing acute attacks? Action plans?  Advice on living with asthma? |
|  | Establish what supported self management means to group/interviewee but if they have a different understanding, need to establish a common understanding before moving to the next question. | |
| 5-10 mins | **Fitting self-management into the routines of practice** | What is the current policy in your practice? (Is SMP something that is given to patients here and how readily is it available?  Routines? (What, how often)  Role of others in team? (Are others/should others be involved? Who initiate SMP? Who review SMP? Specifically ask about GPs – if see a patient for exacerbation will they provide/review SMP?)  Skills/training: How do you go about finding information if you identified lack of knowledge, what type of training do you normally access (internally and externally)?  What resources do they have/use? (time/available PAAPs)  Priority of asthma SM in the practice and individually |
| 5 mins | **Barriers**  We already know about the barriers so keep this brief.  On a practical level what are the barriers to self-management in your practice? | Perceptual? Waste of time? Only suitable for some people?  No action plan (or can’t find them)? Worried about patients having emergency supplies of drugs?  No time/resources to provide the (accessible/flexible) support? |
| Most Time | **Practical Strategies**  Education: We are planning to develop an educational intervention, how should we go about it, what would work best for practices? nurses and doctors  What do they think will help them use SMP with patients more regularly, what will make management easier (what should prompt/ IT technology/web links do to help you use SMPS and manage patients asthma)?  How would these fit in with the routines of the practice? | Computer reminders  Easier access to action plans (Format? Digital?)  Apps: Patient access to SMP on an app- would that be suitable to your patient group/ which patient group are likely less likely to use (ask also about parents?)  ‘Advertising’ action plans: How else you may promote the use of SMP- some surgeries advertise on electronic boards, web sites, printed information on prescription to remind them that they need to have a review of their action plan. Would that work in your surgery?  What about dedicated web page where patients can log in confidentially, review their action plan? Cloud-based records? Patient access to EHR?  Flexible access to professional advice? On- line appointments/on day telephone consultations?  Telephone? E-mail? Helpline? Texts? For contacting patients- have they trialled this in their practice, did it work, will they be interested to try? Can it be used for a 2- way communication? |
|  | What do you think of these ideas? | We will introduce suggestions from literature, and as the data collection and analysis proceeds, from previous focus groups and interviews, professional/manager focus groups and interviews. |
|  | How keen would you be to see supported self-management implemented as a normal way of working? | Any more ideas about how this could be achieved within the routines of primary care? |
