# Supplementary material for: Exploring the perspectives of clinical professionals and support staff on implementing supported self-management for asthma in UK general practice: an IMP2ART qualitative study
Source: NPJ Prim Care Respir Med. 2017 Jul 18;27:45. doi: 10.1038/s41533-017-0041-y (PMC5515882; doi:10.1038/s41533-017-0041-y)
Supplement: Supplementary file 2 — Appendix 2. Coding framework [file 41533_2017_41_MOESM2_ESM.docx]

Primary and Secondary Codes Derived from the Data

| **Primary Codes** | **Secondary Codes** |
| --- | --- |
| **1. Definition of Supported Self Management** |  |
| **2. Organisational Delivery Systems** | Annual Review |
|  | Duplication of Work |
|  | Emergency Appointments |
|  | Exacerbations |
|  | Flexible Appointments |
|  | Hospital Discharge |
|  | Mode of Access |
|  | Non-Attenders |
|  | Opportunistic Review |
|  | Organisational Barriers |
| **3. People** |  |
| **3.1 Patients** | Attitude |
|  | Behavioural Change |
|  | Challenging Patients |
|  | Co-Morbidities |
|  | Confidence |
|  | Empowerment |
|  | Engagement |
|  | Family Involvement |
|  | Individualism |
|  | Knowledge |
|  | Language Barriers |
|  | Peer Support |
|  | Resistant to Change |
|  | Sense of Responsibility |
| **3.2 Staff** | Attitude to Supported Self-Management |
|  | Buy-in |
|  | Confidence (self) |
|  | Confidence in Patients |
|  | Continuity |
|  | Entrenched Culture |
|  | Patient Centred Care |
|  | Priorities |
|  | Roles and Responsibilities |
|  | Trust in Patients |
| **4. Structuring Devices/Technology** | Access to Secondary Care Advice |
|  | Advertising |
|  | Clinical Templates - current use |
|  | Clinical Templates - improvements |
|  | Computer Prompts |
|  | Confidentiality and Access to Records |
|  | Digital Solutions |
|  | Educating and Including Other Healthcare Professionals |
|  | Integration with Electronic Health Records |
|  | Media Campaign |
|  | Personal Asthma Action Plans - improvements |
|  | Personal Asthma Action Plans - inflexibility |
|  | Web Based |
| **5. Education and Training** | Patients |
|  | Professionals |
| **6. Emerging and Other Themes** | Misdiagnosis |
|  | Quality and Outcome Framework |
